# High-Frequency Three-Dimensional Lumen Volume Ultrasound Is a Sensitive Method to Detect Early Aneurysmal Change in Elastase-Induced Murine Abdominal Aortic Aneurysm

**DOI:** 10.1055/s-0041-1731404

**Published:** 2021-12-28

**Authors:** Mohammed A. Waduud, Parkavi Kandavelu, Melanie Reay, Katherine Paradine, David J. A. Scott, Marc A. Bailey

**Affiliations:** 1 Leeds Institute for Cardiovascular and Metabolic Medicine, School of Medicine, University of Leeds, Leeds, United Kingdom; 2 Leeds Vascular Institute, Leeds General Infirmary, Leeds Teaching Hospitals NHS Trust, Leeds, United Kingdom

**Keywords:** abdominal aortic aneurysm, imaging, ultrasound, modeling, growth

## Abstract

**Objective** The aim of this study was to investigate the reproducibility of anterior–posterior diameter (APd _max_ ) and three-dimensional lumen volume (3DLV) measurements of abdominal aortic aneurysms (AAA) in a classical murine AAA model. We also compared the magnitude of change in the aortic size detected with each method of assessment.

**Methods** Periadventitial application of porcine pancreatic elastase (PPE AAA) or sham surgery was performed in two cohorts of mice. Cohort 1 was used to assess for observer variability with the APd _max_ and 3DLV measurements. Cohort 2 highlighted the relationship between APd _max_ and 3DLV and changes in AAA detected.

**Results** There was no significant observer variability detected with APd _max_ measurement. Similarly, no significant intraobserver variability was evident with 3DLV; however, a small but significant interobserver difference was present. APd _max_ and 3DLV measurements of PPE AAA significantly correlated. However, changes in the AAA morphology were detected earlier with 3DLV.

**Conclusion** APd _max_ and 3DLV are both reliable methods for measuring an AAA. Both these methods correlate with each other. However, changes in AAA morphology were detected earlier with 3DLV, which is important to detect subtle but important changes to aortic geometry in a laboratory setting. 3DLV measurement of AAA is a simple, reproducible, and comprehensive method for assessing changes in disease morphology.

## Introduction

An abdominal aortic aneurysm (AAA) is a progressive focal aortic dilation. In humans, the maximal anteroposterior aortic diameter (APd _max_ ) on ultrasound (USS) is typically used to quantify aortic size. 1 Murine AAA models (e.g., periadventitial porcine pancreatic elastase [PPE] 2 ) can be used to gain biological insights and trial novel therapeutics in the preclinical laboratory setting. However, while the normal human aortic APd _max_ may be up to 3 cm, the murine aortic APd _max_ maybe up to 60 times smaller. 3 Despite this, the APd _max_ is routinely utilized in preclinical research using murine AAA models. 4 5

While such a measurement can detect the presence or absence of AAA, we hypothesize that it may not be able to accurately detect subtle but important changes to aneurysm geometry which occur in response to manipulation of pertinent genes relating to aneurysm biology or following administration of novel disease-modifying substances. Furthermore, single APd _max_ measurement also neglects the length of the aneurysmal segment and hence may not be the optimal marker of disease severity in the preclinical setting and arguably in the clinical setting. 6 7

Measurement of three-dimensional lumen volume (3DLV) of murine AAA using USS has good correlation with volumetric reconstruction of histological sections through the same aorta. 8 However, this technique requires specialist equipment to capture high-frequency, high-resolution data, and standardization of the region of interest studied and gating for cardiac and respiratory motion. The operator must be able to identify and demarcate the correct aortic segment and must have access to software available to facilitate rapid semiautomated quantification of the aortic volume. The inter- and intraobserver variability of the 3D USS technique remain unreported and the differences in the capability to detect aneurysmal change compared with APd _max_ remains unclear.

The primary aim of this study was to present a standardized method for 3DLV ultrasound of the infrarenal aorta in mice in the context of PPE-induced AAA and to investigate observer reproducibility compared with APd _max_ . Second, we compared the differences in measurements using the two methods for detecting the presence of an AAA using serial scanning following PPE application to the aorta. We also assessed the magnitude of the changes in the AAA detectable with each method of measurement.

## Materials and Methods

### Murine Porcine Pancreatic Elastase Abdominal Aortic Aneurysm Model

Male C57BL6/J mice were purchased from Charles River ( *https://www.criver.com* , United Kingdom) and used for experiments at 12 weeks of age. Animals were housed in GM500 cages (Techniplast, Italy) with a 12-hour light/dark cycle and free access to standard chow diet and triple-filtered drinking water. Each cage contained a dome home and chew sticks as environmental enrichment. The animals were housed at a maximum of five per cage.

Midline laparotomy was performed under recovery isofluorane anesthesia and the aorta exposed using blunt dissection and PPE or saline applied to the aorta for 5 minutes, as described by Bhamidipati et al. 2 The peritoneal cavity was washed out three times with normal saline and the abdominal wall closed in layers with Vicryl sutures. Two independent cohorts of mice (cohort A and cohort B) were used in this study. Cohort A was used to assess observer variability in USS measurements. Cohort B was used to assess single APd _max_ /3D USS measurement sensitivity in detection of aortic dilation.

Animal work was performed in accordance with the UK Animals, Scientific Procedures Act 1986. This study was performed under existing institutional approval (the Home Office Project License PPL: P606320FB). All investigators undertook additional training to obtain Home Office personal animal licenses.

### In Vivo High-Resolution Ultrasound

All mice were prepared by fur removal shaving (Contura, Weller) and deepilation (Veet) of the anterior abdominal wall from the costal margins (superior margin) to the pubis (inferior margin) and midaxillary line to midaxillary line (lateral margins). Ultrasound was performed with a Vevo2100 high-frequency preclinical micro USS (Visualsonics, the Netherlands). This included an automatic motor, an MS-550D probe (40 MHz frequency), a heated platform with electrocardiogram and respiratory recording, and a thermometer. Transverse imaging was performed on a layer of aquasonic gel (Parker Labs, the Netherlands). All imaging was gated for respiration and triggered at 50 ms after the r wave, as this corresponded to peak dilation in the infrarenal abdominal aorta. Mice were imaged under recovery isoflurane anesthesia.

**Fig. 1:**
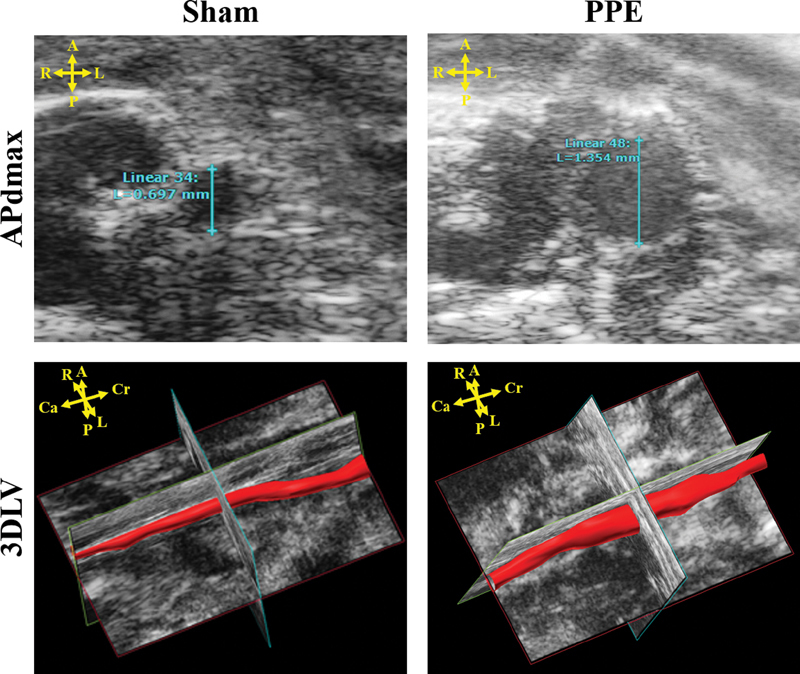
Example images from the Vevo2100 demonstrating anterior–posterior diameter (APd _max_ ) and three-dimensional lumen volume (3DLV) measurements in sham control and periadventitial porcine pancreatic elastase (PPE) treated mice. A, anterior, Ca, caudal; Cr, cranial; L, left; P, posterior; R, right.

The APd _max_ (inner-to-inner) was calculated from a single electrocardiography-gated Kilohertz Visualization (EKV) recording which was made in the brightness (“B”) mode at the largest section of the aorta ( Fig. 1 ). The 3DLV was calculated from serial transverse images recorded along an 11.96-mm segment caudally from the left renal artery (157 frames at 0.076 mm intervals). These images were reconstructed into a 3D image by the VevoLab (Visualsonics, the Netherlands) software package ( Fig. 1 ).

### Image Segmentation

Image analysis for both the APd _max_ and 3DLV was performed in VevoLab v1.7.0 (Visualsonics, the Netherlands). The inner-to-inner aortic diameter was measured in millimeters from the single slice cross-sectional EKV recording to obtain the APd _max_ . However, to assess the 3DLV, the inner lumen was manually traced every forth slice recorded below the left renal artery. The software subsequently rendered the manual tracings to derive a volumetric output in cubic millimeters (mm ^3^ ).

### Assessment of Observer Variability

To assess for the observer variability, APd _max_ and 3DLV measurements were recorded from imaging performed at day 14 post surgery (i.e., PPE or sham). Two independent trained observers (i.e., observer 1 [O1] and observer 2 [O2]) recorded measurements blinded to the measurements of the other and to the surgery performed on the animal (sham or PPE). Both observers were doctoral students who were trained by an independent expert coinvestigator with at least 5 years' experience in murine and human USS. O1 had a basic science background and O2 was a resident in vascular surgery with prior experience in USS. O2 measured the images twice (O2a and O2b) blinded to the results from his previous measurement to determine intraobserver variability. Images were presented to each observer in a random order.

### Detection of Aneurysm Growth

For comparative analysis, of APd _max_ and 3DLV measurements, imaging was performed preoperatively and then every 3 to 5 days postoperatively for 14 days in an independent cohort of mice. Single observer (O2) measurements were utilized as this was likely to reflect routine laboratory practice. At each time point, APd _max_ measurements were compared with 3DLV measurements of both sham and PPE mice. Percentage change over time in aortic dilation (compared with baseline) detected by each measurement technique was assessed.

### Statistical Analysis

Differences in observer measurements were evaluated using Bland–Altman plots with limits of agreement (LOA), Pearson's correlation, and two-sample *t* -tests. The relationship between APd _max_ and 3DLV measurements was assessed using Pearson's correlation and linear regression analysis. Differences in measurements between the groups (i.e., sham and PPE) at time points were evaluated using two-sample tests. Measurements were reported as a mean ± standard deviation (SD). Percentage change detected with each technique was evaluated using scatter plots and the lines of best fit.

Statistical analyses were performed using Minitab (Minitab, PA). A *p* -value < 0.05 was considered significant.

## Results

In total, cohort A consisted of 10 mice (7 PPE and 3 sham) and cohort B of 14 mice (9 PPE and 5 sham). There was no case of aneurysm rupture or death during the study.

### Observer Variability

**Fig. 2:**
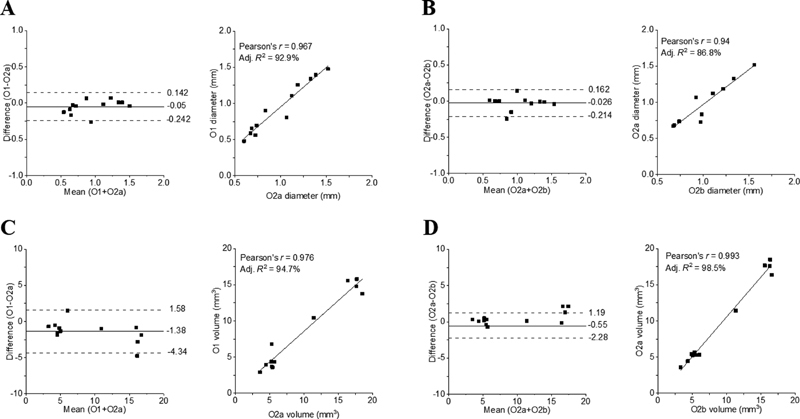
Bland–Altman plots and correlation analysis of: ( **A** ) interobserver variations in anterior–posterior diameter (APd _max_ ) measurements, ( **B** ) intra-observer variations in APd _max_ measurements, ( **C** ) interobserver variation in three-dimensional lumen volume (3DLV) measurements and ( **D** ) intraobserver variation in 3DLV measurements. Adj., adjusted; O1, observer 1; O1a, first image by observer 1; O2, observer 2; O2a, first image by observer 2; O2b, second image by observer 2.

There were no significant interobserver differences (mean difference: −0.05, LOA: 0.14–0.24, *p* = 0.100; Fig. 2A ) or intraobserver differences (mean difference: −0.03 mm, LOA: 0.16 to −0.21, *p* = 0.36; Fig. 2B ) in the measurement of the APd _max_ . Despite a strong positive correlation ( *r* = 0.98, *p* < 0.001), there was a small but significant difference in the interobserver variability in 3DLV measurements (mean difference: −1.38, LOA: 1.58 to −4.34, *p* = 0.008; Fig. 2C ). There were also no significant intraobserver differences in 3DLV measurements (mean difference: −0.55, LOA: 1.19 to −2.28, *p* = 0.053; Fig. 2D ). Measurements of both the APd _max_ and 3DLV were also well correlated between O1 and O2 ( Fig. 2 ). Repeated measurements of the APd _max_ and 3DLV by O2 (i.e., O2a and O2b) were also well correlated ( Fig. 2 ).

### Measurement Techniques

**Fig. 3:**
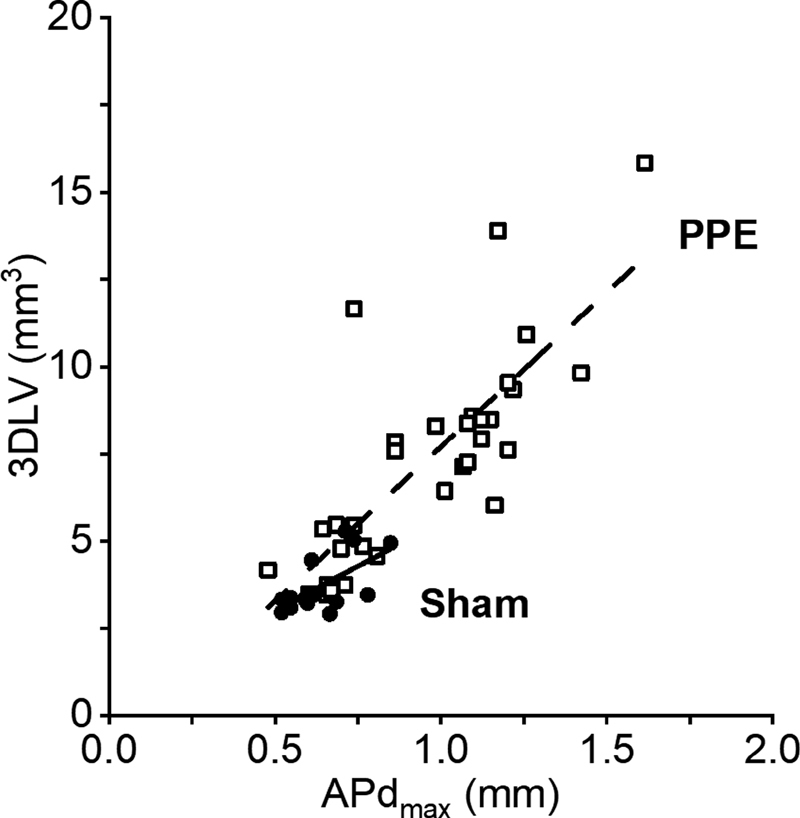
Relationship between anterior–posterior diameter (APd _max_ ) and three-dimensional lumen volume (3DLV) measurements in mice treated with periadventitial porcine pancreatic elastase (PPE; open squares) and sham controls (closed circles). The solid lines represent the associations between the two measurements for the sham ( *r* = 0.24, adjusted *R* ^2^ = − 0.06, *p* = 0.5) and PPE groups ( *r* = 0.70, adjusted *R* ^2^ = 0.46, *p* < 0.001).

We observed significant positive correlations between APd _max_ and 3DLV measurements in cohort B overall ( *r* = 0.83, *p* < 0.001). This trend remained evident in subanalysis of aortic measurements in mice which had undergone PPE surgery ( *r* = 0.70, *p* < 0.001; Fig. 3 ). However, this correlation was not apparent in measurements from mice which had undergone sham surgery only ( *r* = 0.24, *p* = 0.5; Fig. 3 ).

**Fig. 4:**
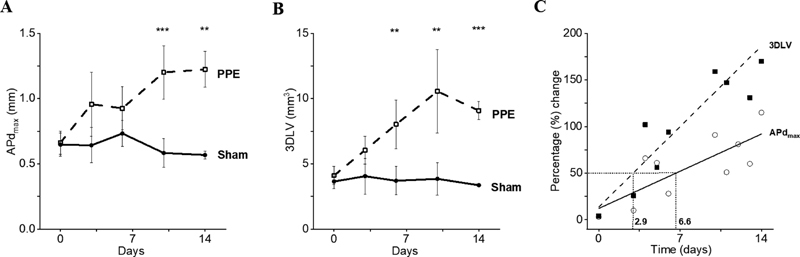
Differences in aortic measurements using anterior–posterior diameter (APd _max_ ) ( **A** ) and three-dimensional lumen volume (3DLV) ( **B** ) in sham controls (closed circles) and periadventitial porcine pancreatic elastase (PPE) treated mice (open squares). Percentage change in aortic size detected with time ( **C** ) with APd _max_ (open circles) compared with 3DLV (closed squares). The time taken to detect a 50% increase in abdominal aortic size is shown with the dotted lines.

Both measurements, APd _max_ and 3DLV, were able to detect the presence of AAA at the standard time point of the model (i.e., 14-day postoperatively; Fig. 4 ). Absolute mean differences between the sham and PPE groups were 0.66 ± 0.11 mm for APd _max_ and 5.72 ± 0.56 mm ^3^ for 3DLV. However, the 3DLV technique was able to identify a statistically significant aneurysmal dilatation at an earlier time point than the APd _max_ method ( Fig. 4A and B ). Furthermore, the percentage increase in sequential aortic measurements compared with baseline over the course of the 14 days was approximately two-fold greater using 3DLV measurements compared with APd _max_ ( Fig. 4C ). For example, a 50% increase in aortic measurements was seen after approximately 3 days using 3DLV measurements compared with nearly 7 days using APd _max_ measurements ( Fig. 4C ). This was also reflected in the different rates of growth detected (i.e., 3DLV slope 12.3 versus APd _max_ slope 5.7; Fig. 4C ).

## Discussion

We describe a standardized method for APd _max_ and 3DLV assessment of the abdominal aorta in the PPE murine model of AAA using a Vevo2100 high-resolution preclinical ultrasound system. 3DLV provides a simple, reproducible, and comprehensive measurement of geometric changes in the infrarenal aorta in mice comparable to traditional APd _max_ measurement. 9 10 However, 3DLV can detect aneurysmal changes earlier than APd _max,_ and the magnitude of the detectable difference is also larger. This likely relates to the capture of the entire length of the aneurysmal segment when utilizing 3DLV measurement, rather than a single slice which may fail to capture subtle changes.

This is supported by the data from contemporary studies, which demonstrated that two-dimensional (2D) USS techniques overlook regional differences evident with volumetric USS quantification. 11 Furthermore, 3DLV measurement of murine aorta has been demonstrated to highly correlate with more sensitive modalities, such as computer tomography and magnetic resonance imaging, as well as histological assessment of aneurysmal aorta. 8 12 13 Despite these observations, 2D USS assessment of murine aorta remains the dominant strategy. 9 10 The results of our study may help preclinical researcher interpret and compare the results between studies utilizing the two differing measurement techniques.

Similar to our method, Soepriatna et al 11 utilized cardiac and respiratory gating capturing multiple images over the aneurysmal segment of the aorta. However, they utilized the angiotensin II (AngII) AAA murine model. However, direct comparisons between 2D and volumetric measurements of the murine aortic were not reported as described in our study. Importantly, they highlighted volumetric USS to take between two and four times longer than 2D measurements, which may explain the reluctance for routine adoption in basic science research.

While strong positive correlations in measurements with each method between observers were seen, there were significant absolute differences in the 3DLV measurements between observers. To calculate the 3DLV, the observer is required to delineate the aortic lumen from the surrounding anatomy. This method is, therefore, subject to the operator's interpretation of the grayscale sonographic images as to exactly where this boundary exists. Small differences in the positioning of the measuring calibers between observers are then incurred across multiple frames of analysis. Despite this small potential for absolute differences, it is reassuring that the observations are well correlated between observers. This suggests that observers can reliably identify the same boundaries consistently across different animals, but that there will still be subtle differences between different observers. Therefore, we recommend single observer measurement, blinded to treatment allocation, of all imaging in a single experiment.

We evaluated 3DLV measurements in only the periluminal PPE AAA model based on the expertise of our group. However, the technique can also be applied to the intraluminal PPE model or the CaCl _2_ models using the protocol described. For the AngII AAA model, 14 where the aneurysm has a propensity to form in the suprarenal segment of the abdominal aorta, the same approach could be adapted by setting the region of interest to capture images 12-mm cranially starting from the right renal artery.

### Limitations of the Study

We solely utilized male mice, as they were more likely to develop an AAA and also due to the high prevalence of AAA disease in the male population. 15 16 We did not feel it necessary to validate APd _max_ and 3DLV in the AngII AAA model or female mice, as the underlying mathematical relationships between the measurement techniques should theoretically be the same. However, based on our findings, we would advocate the use of 3DLV in AAA murine experimentation utilizing female mice, as it may facilitate the detection of subtle changes within the aorta.

## Conclusion

In conclusion, the ability to detect changes in the abdominal aorta with 3DLV measurements makes this technique ideally suited to evaluate gene or treatment effects which modulate AAA development and progression in the preclinical setting. 3DLV measurement may detect clinically significant changes that would be plagued by type-I error when using APd _max_ .

## References

[1] Multicentre Aneurysm Screening Study Group Ashton H A Buxton M J Day N E The Multicentre Aneurysm Screening Study (MASS) into the effect of abdominal aortic aneurysm screening on mortality in men: a randomised controlled trial Lancet 2002 360(9345):1531 1539 12443589 10.1016/s0140-6736(02)11522-4

[2] Bhamidipati C M Mehta G S Lu G Development of a novel murine model of aortic aneurysms using peri-adventitial elastase Surgery 2012 152 02 238 246 22828146 10.1016/j.surg.2012.02.010 PMC3601193

[3] Guo X Kono Y Mattrey R Kassab G S Morphometry and strain distribution of the C57BL/6 mouse aorta Am J Physiol Heart Circ Physiol 2002 283 05 H1829 H1837 12384460 10.1152/ajpheart.00224.2002

[4] Martin-McNulty B Vincelette J Vergona R Sullivan M E Wang Y-X Noninvasive measurement of abdominal aortic aneurysms in intact mice by a high-frequency ultrasound imaging system Ultrasound Med Biol 2005 31 06 745 749 15936490 10.1016/j.ultrasmedbio.2005.02.012

[5] Barisione C Charnigo R Howatt D A Moorleghen J J Rateri D L Daugherty A Rapid dilation of the abdominal aorta during infusion of angiotensin II detected by noninvasive high-frequency ultrasonography J Vasc Surg 2006 44 02 372 376 16890871 10.1016/j.jvs.2006.04.047

[6] Ghulam Q M Kilaru S Ou S-S Sillesen H Clinical validation of three-dimensional ultrasound for abdominal aortic aneurysm J Vasc Surg 2020 71 01 180 188 31204220 10.1016/j.jvs.2019.03.066

[7] Long A Rouet L Debreuve A Abdominal aortic aneurysm imaging with 3-D ultrasound: 3-D-based maximum diameter measurement and volume quantification Ultrasound Med Biol 2013 39 08 1325 1336 23743100 10.1016/j.ultrasmedbio.2013.03.008

[8] Goldberg A Pakkiri P Dai E Lucas A Fenster A Measurements of aneurysm morphology determined by 3-d micro-ultrasound imaging as potential quantitative biomarkers in a mouse aneurysm model Ultrasound Med Biol 2007 33 10 1552 1560 17602826 10.1016/j.ultrasmedbio.2007.04.003

[9] Sawada H Chen J Z Wright B C Moorleghen J J Lu H S Daugherty A Ultrasound imaging of the thoracic and abdominal aorta in mice to determine aneurysm dimensionsJ Vis Exp 2019;145 10.3791/59013 PMC6594159 30907888

[10] Cao R Y Amand T Ford M D Piomelli U Funk C D The murine angiotensin II-induced abdominal aortic aneurysm model: rupture risk and inflammatory progression patterns Front Pharmacol 2010 9 1 9 10.3389/fphar.2010.00009 PMC3112241 21713101

[11] Soepriatna A H Damen F W Vlachos P P Goergen C J Cardiac and respiratory-gated volumetric murine ultrasound Int J Cardiovasc Imaging 2018 34 05 713 724 29234935 10.1007/s10554-017-1283-z PMC5889723

[12] Jansen C HP Reimann C Brangsch J Botnar R M Makowski M R In vivo MR-angiography for the assessment of aortic aneurysms in an experimental mouse model on a clinical MRI scanner: comparison with high-frequency ultrasound and histology PLoS One 2017 12 06 e0178682 28582441 10.1371/journal.pone.0178682 PMC5459432

[13] Trachet B Fraga-Silva R A Piersigilli A Dissecting abdominal aortic aneurysm in Ang II-infused mice: suprarenal branch ruptures and apparent luminal dilatation Cardiovasc Res 2015 105 02 213 222 25538157 10.1093/cvr/cvu257

[14] Daugherty A Cassis L A Mouse models of abdominal aortic aneurysms Arterioscler Thromb Vasc Biol 2004 24 03 429 434 14739119 10.1161/01.ATV.0000118013.72016.ea

[15] EVAR-1, DREAM, OVER and ACE Trialists Powell J T Sweeting M J Ulug P Meta-analysis of individual-patient data from EVAR-1, DREAM, OVER and ACE trials comparing outcomes of endovascular or open repair for abdominal aortic aneurysm over 5 years Br J Surg 2017 104 03 166 178 28160528 10.1002/bjs.10430 PMC5299468

[16] Daugherty A Manning M W Cassis L A Angiotensin II promotes atherosclerotic lesions and aneurysms in apolipoprotein E-deficient mice J Clin Invest 2000 105 11 1605 1612 10841519 10.1172/JCI7818 PMC300846

